# Changes in hunting season regulations (1870s–2019) reduce harvest exposure on greater and Gunnison sage-grouse

**DOI:** 10.1371/journal.pone.0253635

**Published:** 2021-10-05

**Authors:** Jonathan B. Dinkins, Courtney J. Duchardt, Jacob D. Hennig, Jeffrey L. Beck

**Affiliations:** 1 Department of Animal and Rangeland Sciences, Oregon State University, Corvallis, Oregon, United States of America; 2 Department of Ecosystem Science and Management, University of Wyoming, Laramie, Wyoming, United States of America; Sichuan University, CHINA

## Abstract

Hunter harvest is a potential factor contributing to population declines of sage-grouse (*Centrocercus spp*.). As a result, wildlife agencies throughout western North America have set increasingly more conservative harvest regulations over the past 25 years to reduce or eliminate hunter success and concomitant numbers of harvested greater (*C*. *urophasianus*) and Gunnison (*C*. *minimus*) sage-grouse. Sage-grouse hunting has varied widely over time and space, which has made a comprehensive summary of hunting management challenging. We compiled data on harvest regulations among 11 western U.S. states and 2 Canadian provinces from 1870–2019 to create a timeline representative of hunting regulations. We compared annual harvest boundaries and area-weighted average hunting regulations, 1995–2018, relative to administrative boundaries and areas of high probability of sage-grouse occupation. We also summarized estimated numbers of birds harvested and hunters afield, 1995–2018, across both species’ ranges. From 1995–2018, there was a 30% reduction in administrative harvest boundaries across the greater sage-grouse range compared to a 16.6% reduction in area open to harvest within 8 km from active leks. Temporary closures occurred in response to wildfires, disease outbreaks, low population numbers, and two research projects; whereas, permanent closures primarily occurred in small populations and areas on the periphery of the species distribution. Similarly, area-weighted possession limits and season length for greater sage-grouse decreased 52.6% and 61.0%, respectively, while season start date stayed relatively stable (mean start date ~259 [mid-September]). In contrast, hunting of the now federally-threatened Gunnison sage-grouse ended after 1999. While restrictions in harvest regulations were large in area, closures near areas of high greater sage-grouse occupancy were relatively smaller with the same trend for Gunnison sage-grouse until hunting ceased. For greater sage-grouse, most states reduced bag and possession limits and appeared to adhere to recommendations for later and shorter hunting seasons, reducing potential for additive mortality.

## Introduction

Understanding the effects of human harvest on wildlife populations is necessary under any management scenario but is especially critical when managing declining populations or species of conservation concern. This requires knowing how implementation of harvest management influences population growth relative to hunting regulations, which can be informed by how hunting regulations have changed through time and across administrative boundaries. Among North American game species, prairie and shrubland grouse have emerged as a group of special concern within the past few decades [1]. Attwater’s prairie chicken (*Tympanuchus cupido attwateri*) was listed as endangered under the U. S. Endangered Species Act (ESA) in 1967 [2], while lesser prairie chicken (*T*. *pallidicinctus*) and Gunnison sage-grouse (*Centrocercus minimus*) were listed as threatened in the 2010s [3, 4]. Greater sage-grouse (*C*. *urophasianus*) and the Columbian sharp-tailed grouse (*T*. *phasianellus columbianus*) have also been previously considered for federal listing [5, 6], but are not currently under ESA protections. Meanwhile, hunting continues for greater prairie chicken (*T*. *cupido*), greater sage-grouse, and Columbian sharp-tailed grouse, which in consideration of other factors may lead to poorly understood ramifications for long-term species viability [7].

Habitat loss and alteration of remaining sagebrush (*Artemisia* spp.) habitat have been the greatest drivers of greater and Gunnison sage-grouse declines in the past 100 years [8–12]. The extent of greater and Gunnison sage-grouse habitat has declined by ~44% and ~90%, respectively, since pre-settlement [13], and abundance has declined an estimated ~66% since the mid-1960s and ~66–90% since the 1950s, respectively [14–16]. Estimates of Gunnison sage-grouse population decline from 1950–2000 were not from peer-reviewed analyses; regardless the decline of Gunnison sage-grouse has been significant [14]. In addition, Davis et al. [11] report average growth rates for Gunnison sage-grouse were near 1.0 or <1.0 from 1996–2012. Specific land uses contributing to this decline include agricultural practices [17–20], exurban development [21], energy development (e.g., [19, 22, 23]), and conifer encroachment [10, 12, 20]. Invasion of non-native cheatgrass (*Bromus tectorum*) has increased fire frequency especially in the Great Basin, further threatening sagebrush ecosystems [24–26]. Disease has also been a contributing factor in greater sage-grouse decline in some regions with outbreaks of West Nile virus leading to a 25% decrease in survival in certain populations [27]. Beyond habitat loss and direct mortality, human development has led to reduced genetic diversity via habitat fragmentation [28], reduced breeding success due to reduced concealment opportunities [29], and increased abundance of predators associated with structures and roads [30–34].

This suite of issues co-occurs with continued harvesting of greater sage-grouse throughout most of its range [7], while hunting was eliminated for Gunnison sage-grouse after sage-grouse were split into two species in 2000 [14]. However, hunting was not listed as a major driver of declines in the 2015 listing decision for greater sage-grouse [5]. Although numerous studies indicate that greater sage-grouse populations likely experience compensatory mortality in response to hunting [35–38], some studies have identified patterns consistent with additive mortality [39–41]. These conflicting findings encourage further exploration of agency adjustments to harvest management for greater sage-grouse.

Most harvest studies and summaries of harvest management have focused on specific populations of greater sage-grouse [36, 39]. This, in concert with the fact that currently greater sage-grouse hunting is adaptively managed by state wildlife agencies, makes it difficult to see the full pattern and balance of management objectives for grouse in terms of harvest management across the range of this sensitive species. We summarized the history of harvest management for both greater and Gunnison sage-grouse throughout their ranges with the goals of 1) describing the history of changes to harvest management across both ranges of sage-grouse, especially over the past ~150 years, 2) quantifying specific changes to bag limits, season length and timing, and closures in each of the 11 states and 2 provinces where sage-grouse currently occur during the past 25 years, and 3) comparing bag limit, season length, and area open to hunting during the past 25 years between administrative harvest boundaries and current sage-grouse range.

### Study area

**Fig 1:**
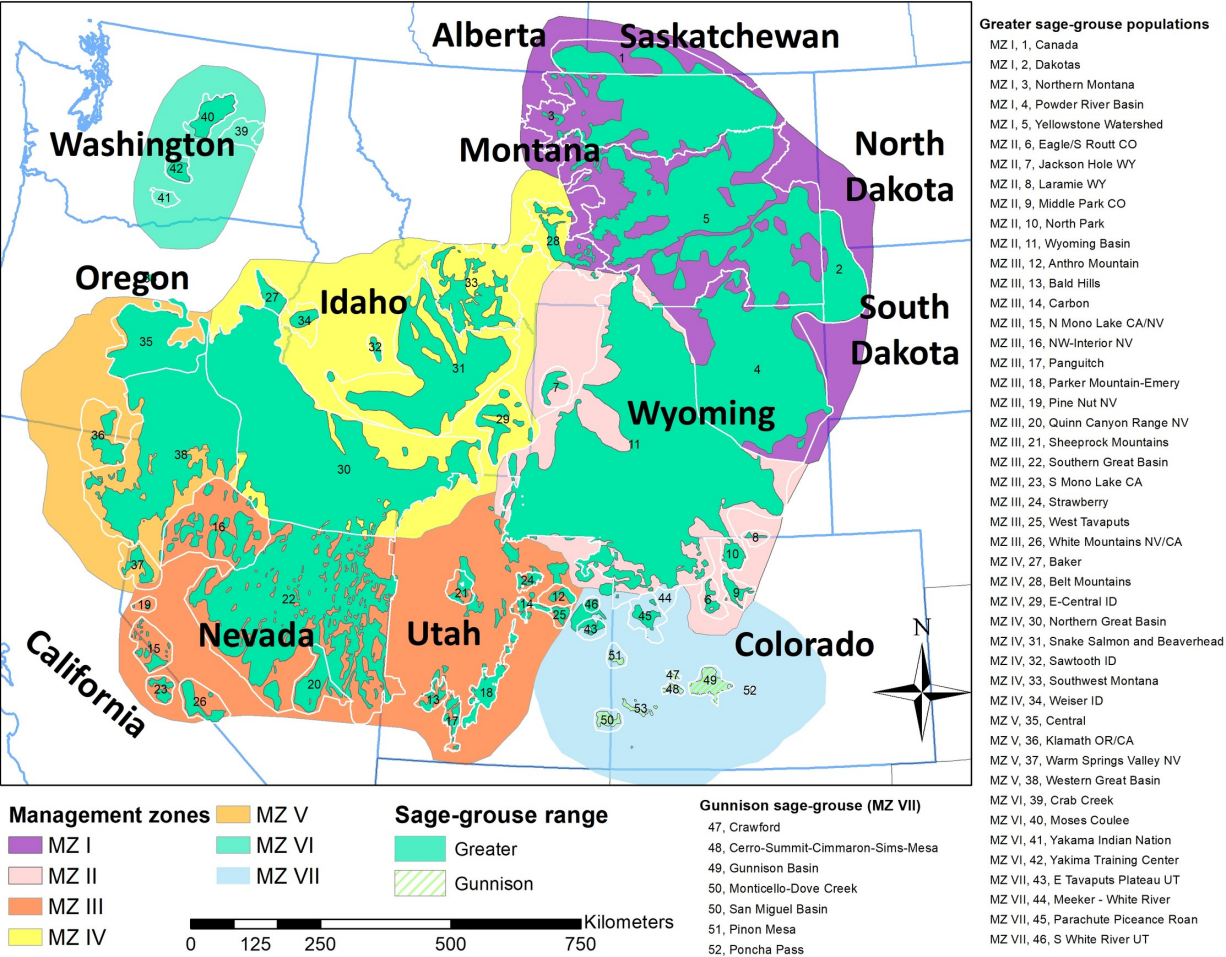
Assessment region, encompassing eleven states and two provinces stratified as seven management zones, and the extant range of both the greater and Gunnison sage-grouse. Population names were defined in USFWS listing documents [4, 5].

Our study covered the entire extant range of greater and Gunnison sage-grouse, and included 11 western U.S. states (California, Colorado, Idaho, Montana, Nevada, North Dakota, Oregon, South Dakota, Utah, Washington, and Wyoming) and 2 Canadian provinces (Alberta and Saskatchewan) with Gunnison sage-grouse occurring within southeastern Utah and south-central Colorado (Fig 1). We obtained season closure information ~1870–2019 and specific regulations, estimated numbers of harvested birds, and hunters afield for 1995–2018 from state and provincial management agencies including Alberta Environment and Parks; California Department of Fish and Wildlife; Colorado Parks and Wildlife; Idaho Fish and Game Department; Montana Fish, Wildlife, and Parks; Nevada Department of Wildlife; North Dakota Game and Fish Department; Oregon Department of Fish and Wildlife; Saskatchewan Ministry of Environment; South Dakota Game, Fish, and Parks; Utah Division of Wildlife Resources; Washington Department of Fish and Wildlife; and Wyoming Game and Fish Department. We digitized administrative harvest boundaries to the smallest harvest unit available from all management agencies using ArcMap version 10.2 (ESRI, Redlands, CA). Harvest boundaries from all harvest units were annotated with the corresponding legal area open to hunting, bag/possession limits, season length (number of days and weekends), and Julian date of the opening day for each year (Box 1).

**Box 1. Harvest background and terminology.**

**Table:** 

*A comprehensive summary of the history of sage-grouse harvest regulations is challenging because management efforts in the 11 states and 2 provinces within the current species’ range vary widely*. *Until the early 1900s harvest was largely unregulated (Hornady 1916)*, *although some legislation was passed in the late 19*^*th*^*Century (e*.*g*., *Colorado 1877 [Rogers 1964])*, *Montana 1870 [Wallestad 1975])*. *Later into the 1900s regulations increased at the state level*. *As compared to other avian game species*, *upland game birds are subject to far less federal regulation in the United States*. *For instance*, *non-game birds and waterfowl are protected under the****Migratory Bird Treaty Act (1918)****while waterfowl conservation is also supported by the****Duck Stamp Act (1934)***. *Like all game species in the United States*, *sage-grouse conservation is funded to some extent under the Pittman-Robertson Act of 1937 (the federal tax on hunting equipment)*. *Gunnison sage-grouse have been listed as threatened since 2014 under the****Endangered Species Act (1973****)*, *but the greater sage-grouse (and many other upland gamebirds) experience a relatively greater proportion of state regulation as compared to other bird species within the United States (although we note hunting of grouse in Canada is regulated under a federal permit)*. *As such*, *an understanding of the specific terminology associated with hunting regulations*, *and the variation of these regulations among states*, *is critical*.	**Definitions** **Daily bag limit**: maximum number of game birds or small game animals that may be legally taken in a single day. **Possession limit**: maximum number of game birds or small game animals that may legally be in possession following a set number of daily bag limits. Wildlife in transit or storage shall be considered in possession. **Emergency closures:** Wildlife commissioners may institute emergency closure by regulation to shorten hunting seasons in areas if a harvest quota is reached or if emergency situations warrant closure. **Hunt type:** General license (over-the-counter purchase) or Permit. Permits that include a set number of tags in each harvest management area with the number of tags set by wildlife commissioners. Generally limited except in the case of Utah for greater sage-grouse from 2000–2001. Permits can either be for all upland game or species-specific, as in California, Oregon, and Utah. **Hunt area:** The geographical area designated for hunting a particular species, with particular methods, during established hunting seasons. **First weekend:** Hunting seasons generally begin on a weekend, although in some states and years opening day occurs on a weekday, effectively reducing the number of hunters afield as well as season length.

## Materials and methods

We conducted a thorough literature search and obtained season closure documentation from management agencies in all 11 western U.S. states and 2 Canadian provinces from 1870s–2019. We used this information to generate an inclusive timeline detailing the history of sage-grouse hunting. We constructed our timeline to be as detailed as possible relative to sage-grouse populations and sub-divided it by state and provincial boundaries from historical documentation.

**Fig 2:**
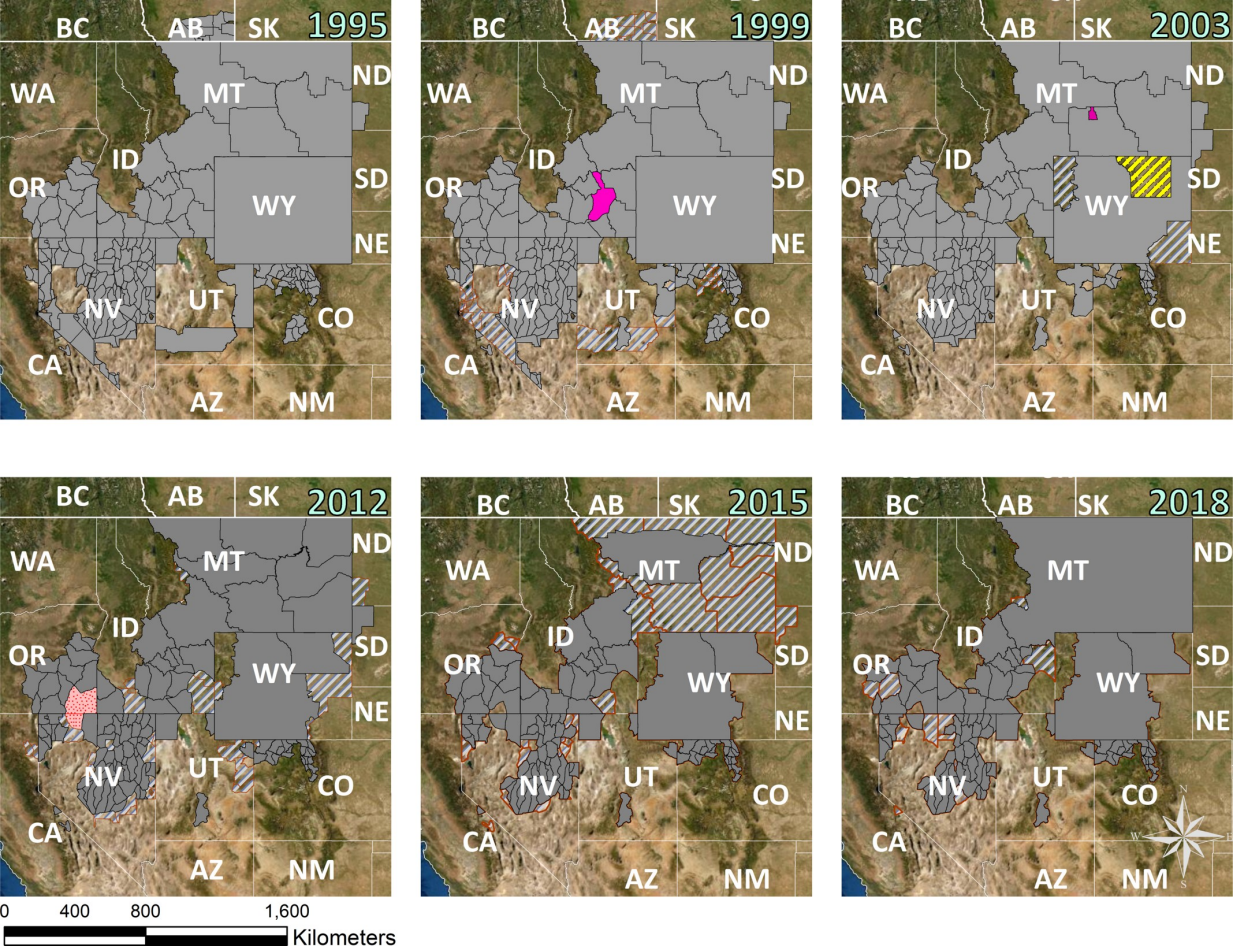
Greater and Gunnison sage-grouse hunt units in the United States and Canada in 1995, 1999, 2003, 2012, 2015, and 2018 within the 1995–2019 study period. Closure types are indicated as follows: small or peripheral populations (grey striped), closures due to federal listing of Gunnison sage grouse (green), experimental closures (pink), closures due to fire (dotted red) and closures related to West Nile Virus (yellow striped).

We provide descriptions and comparisons of legal hunting area, bag and possession limits, season length (number of days and weekends), and season start date, 1995–2018. Season start dates are enumerated in Table 1. For harvest boundaries, the area legally open for greater sage-grouse hunting (administrative boundaries) was compared to the area of legal harvest where greater sage-grouse were most likely to be exposed to hunters. We defined the area where greater sage-grouse were most likely exposed to hunters as 8-km (documented distance where most annual activity for greater sage-grouse occurs) around all active sage-grouse leks (lek count of ≥2 birds any year 1995–2018) on Doherty et al. [42], Fedy et al. [43], and Coates et al. [44]. We calculated the percent decline of area within harvest boundaries (“administrative” and “within 8 km of leks”) for each year based on percent difference compared to 1995. We chose to start this season regulation assessment in 1995 because documentation of changes to hunting regulations by state agencies were more easily obtainable after1995, and there has been increased concern over the conservation status of sage-grouse since 1995. We used area-weighted averages of bag and possession limits, and season length (number of days and weekends) to describe the average harvest effort related to these variables across all sage-grouse populations. Area-weighted averages represented relative harvest effort across different management agency jurisdictions. We area-weighted in two different ways: one based on the total range of sage grouse (“Total range”), and one based on the total range of sage-grouse exposed to hunting pressure (“Harvest range”). The latter range was smaller because many smaller populations and areas on the periphery of the greater sage-grouse range were closed to hunting. We also quantified the legal area open to hunting of Gunnison sage-grouse from 1995–1999, before complete closure of hunting for this species after the hunting season in 1999. Although Gunnison sage-grouse were not recognized as a distinct species until 2000, for these analyses we knew the exact areas where they had existed throughout the assessment period, and that they did not overlap spatially with greater sage-grouse. We did not go into greater detail comparing administrative boundaries relative to areas where Gunnison sage-grouse were exposed to hunting, because both states (Colorado and Utah) with Gunnison sage-grouse completely closed hunting across the species range (Fig 2).

**Table 1:** Area-weighted average daily bag (Bag) and possession (Poss) limits for states and provinces open to hunting greater sage-grouse between 1995–2018. Blank cells indicate closures in given years. Where bag or possession limits varied within a state, minimum and maximum values are presented in parentheses.

	ALB		CA		CO		ID		MT		ND		NV		OR		SD		UT		WY	
Year	Bag	Poss	Bag	Poss	Bag	Poss	Bag	Poss	Bag	Poss	Bag	Poss	Bag	Poss	Bag	Poss	Bag	Poss	Bag	Poss	Bag	Pos
1995	1	2	1.5 (1–2)	1.5 (1–2)	1.34 (1–2)	2.69 (2–4)		6	3	12	1	1	2.02 (2–3)	3.91 (2–6)	2	2	0	0	1.5 (1–2)	3 (2–4)	3	6
1996	0	0	1.5 (1–2)	1.5 (1–2)	1.34 (1–2)	2.69 (2–4)	1.46 (1–2)	2.91 (2–4)	2	6	1	1	2.02 (2–3)	3.87 (2–6)	2	2	0	0	1	2	3	6
1997	0	0	1.5 (1–2)	1.5 (1–2)	1.34 (1–2)	2.69 (2–4)	1.46 (1–2)	2.91 (2–4)	2	6	1	1	2.02 (2–3)	3.05 (2–6)	2	2	0	0	1	2	3	6
1998	0	0	1.5 (1–2)	1.5 (1–2)	2	4	1.46 (1–2)	2.91 (2–4)	2	6	1	1	2.02 (2–3)	3.05 (2–6)	2	2	0	0	1	2	3	6
1999	0	0	1.5 (1–2)	1.5 (1–2)	2	4	1.46 (1–2)	2.91 (2–4)	2	6	1	1	2.03 (2–3)	4.05(4–6)	2	2	0	0	1	2	3	6
2000	0	0	1.5 (1–2)	1.5 (1–2)	2	4	1.46 (1–2)	2.91 (2–4)	3	6	1	1	2.03 (2–3)	4.05 (4–6)	2	2	1	1	1	2	3	6
2001	0	0	1.5 (1–2)	1.5 (1–2)	2	4	1.46 (1–2)	2.91 (2–4)	3	6	1	1	2.02 (2–3)	4.05 (4–6)	2	2	1	1	1	2	3	6
2002	0	0	1.5 (1–2)	1.5 (1–2)	2	4	1.46 (1–2)	2.91 (2–4)	3	6	1	1	2.02 (2–3)	4.05 (4–6)	2	2	1	1	2	2	2	4
2003	0	0	1.5 (1–2)	1.5 (1–2)	2	4	1.46 (1–2)	2.91 (2–4)	3	6	1	1	2.03 (2–3)	4.06 (4–6)	2	2	1	1	2	2	2	4
2004	0	0	1.5 (1–2)	1.5 (1–2)	2	4	1.46 (1–2)	2.91 (2–4)	3	6	1	1	2.03 (2–3)	4.06 (4–6)	2	2	1	1	2	2	2	4
2005	0	0	1.5 (1–2)	1.5 (1–2)	2	4	1.46 (1–2)	2.91 (2–4)	2	4	1	1	2 (2–2)	4	2	2	1	1	2	2	2	4
2006	0	0	1.5 (1–2)	1.5 (1–2)	2	4	1.44 (1–2)	2.89 (2–4)	4	8	1	1	2 (2–2)	4	2	2	1	1	2	2	2	4
2007	0	0	1.5 (1–2)	1.5 (1–2)	2	4	1.27 (1–2)	2.55 (2–4)	2	4	1	1	2.03 (2–3)	4.06 (4–6)	2	2	1	1	2	2	2	4
2008	0	0	1.5 (1–2)	1.5 (1–2)	2	4	1.27 (1–2)	2.55 (2–4)	2	4	0	0	2.03 (2–3)	4.07 (4–6)	2	2	1	1	2	2	2	4
2009	0	0	1.5 (1–2)	1.5 (1–2)	2	4	1.23 (1–2)	2.46 (2–4)	2	4	0	0	2.03 (2–3)	4.07 (4–6)	2	2	1	1	2	2	2	4
2010	0	0	1.5 (1–2)	1.5 (1–2)	2	3.3 (2–4)	1 (1–1)	2	2	4	0	0	2.03 (2–3)	4.07 (4–6)	2	2	1	1	2	2	2	4
2011	0	0	1.5 (1–2)	1.5 (1–2)	2	3.5 (2–4)	1	2	2	4	0	0	2.03 (2–3)	4.07 (4–6)	2	2	1	1	2	2	2	4
2012	0	0	1	1	2	3.5 (2–4)	1	2	2	4	0	0	2.03 (2–3)	4.07 (4–6)	2	2	1	1	2	2	2	4
2013	0	0	1	1	2	3.5 (2–4)	1	2	2	4	0	0	2	4	2	2	0	0	2	2	2	4
2014	0	0	1	1	2	3.5 (2–4)	1	2	2	4	0	0	2	4	2	2	0	0	2	2	2	4
2015	0	0	1	1	2	3.5 (2–4)	1	2	2	4	0	0	2	4	2	2	0	0	2	2	2	4
2016	0	0	1	1	2	3.5 (2–4)	1	2	2	4	0	0	2	4	2	2	0	0	2	2	2	4
2017	0	0	0	0	2	3.52 (2–4)	1	2	2	4	0	0	2	4	2	2	0	0	2	2	2	4
2018	0	0	0	0	2	3.52 (2–4)	1	2	2	4	0	0	2	4	2	2	0	0	2	2	2	4

We also summarized estimated numbers of harvested birds and hunters afield, 1995–2018, based on the mean estimated numbers reported from each management agency (obtained by hunter surveys and check stations) at the spatial resolution of the independent governing body (i.e., state or province). We then stratified these estimated numbers by the relative abundance of sage-grouse within each state. Idaho, Montana, and Wyoming were considered states with a large number of greater sage-grouse; we classified Colorado, Nevada, Oregon, and Utah as medium; and California, North Dakota, and South Dakota as small. Alberta, Saskatchewan, and Washington did not have tabulated numbers for birds harvested or hunters afield because Saskatchewan and Washington did not harvest greater sage-grouse during 1995–2018, and Alberta had missing data for 1995 and no hunting season from 1996–2018. We were able to separate Gunnison sage-grouse harvest numbers from Colorado and Utah sage-grouse harvest data, because the species is spatially distinct from greater sage-grouse; thus, it was easy to discern which species was being harvested in a given management area.

## Results and discussion

### Timeline of hunting restrictions and area boundaries

**Fig 3:**
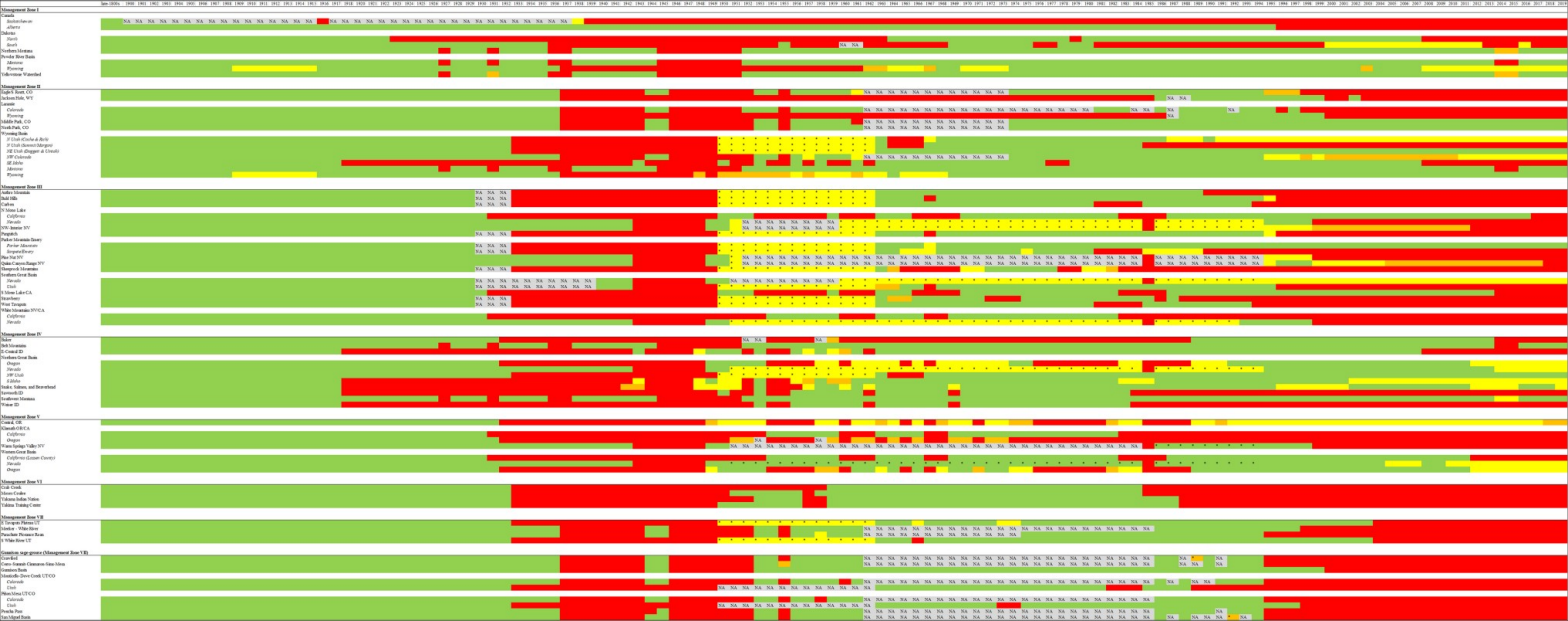
Timeline depicting the harvest history of sage-grouse from 1870s–2019 stratified by sage-grouse Management Zone and greater and Gunnison sage-grouse populations. Information obtained from state and provincial management agencies and 14,35,46,47,48,49,50,51,52,53,54. All populations that crossed state or provincial boundaries were sub-stratified by those legal boundaries. Green depicts years when a population stratified by state was essentially fully open to hunting, yellow depicts partial closures (>50% open but some closure), orange depicted partial closures (<50% open but some legal hunting), red depicted essentially full hunting closures, and grey with NA indicates years where no data was available. This information is also available in Excel format via S1 Table.

Regulated hunting seasons for sage-grouse started in the 1870s for some states with Montana starting in 1870, Colorado in 1877, Idaho in 1900, Wyoming in 1902, Oregon in 1903, and Washington in 1933 [35,45,46,47, 48,49,50,51,52,53,54]. Sage-grouse were commercially harvested in the late-1800s but transitioned to sport hunting as state management agencies implemented hunting regulations. We found all areas were open for hunting prior to 1916 when Saskatchewan was closed to hunting [55]. The 1930s and 1940s were decades with the most area closed to hunting during the past 15 decades (Fig 3). These closures were based on concerns of overharvest resulting in poor population growth across the ranges of greater and Gunnison sage-grouse [53]. The 1970s and 1980s were decades where hunting was open throughout most of the sage-grouse distribution, but season regulations were more conservative than the early 1900s (Fig 3).

**Fig 4:**
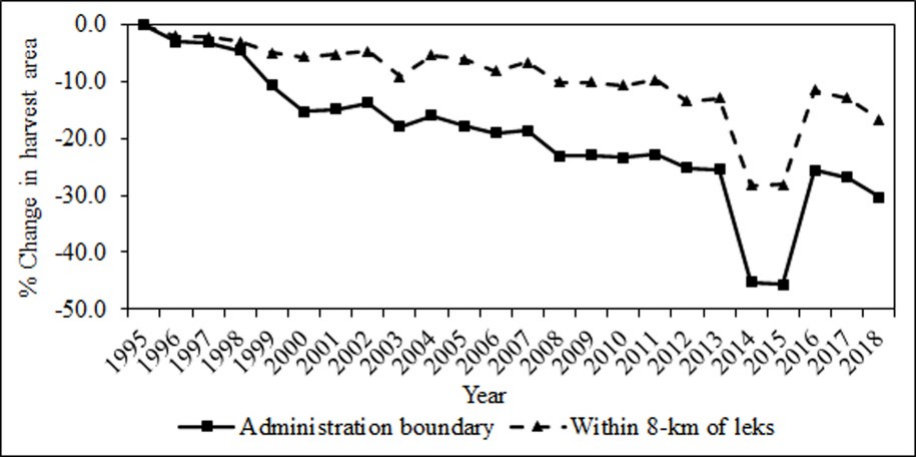
Change in total harvest area for greater sage-grouse across 11 western states and 2 Canadian provinces from 1995–2018, represented by total administrative boundaries (solid) and area within 8 km of known leks (dashed).

**Fig 5:**
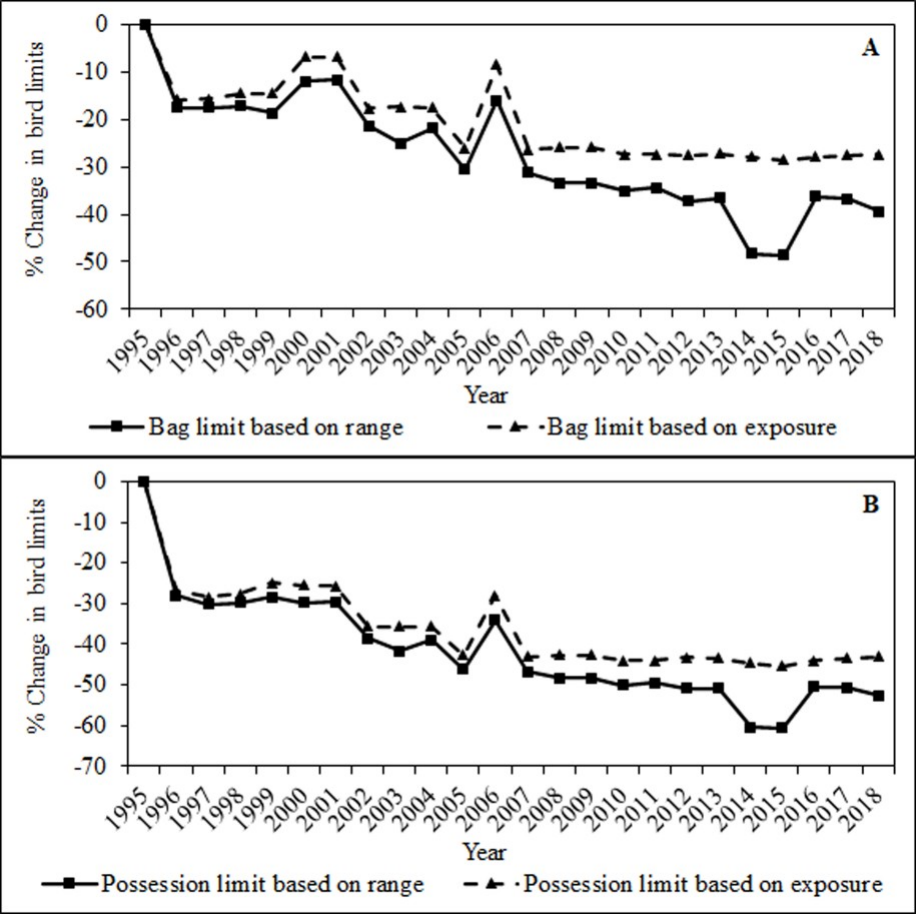
**Change over time in average bag limits (A) and possession limits (B) of greater sage-grouse, 1995–2018**. The solid line represents averaged season length within 8 km of all active greater sage-grouse leks, and the dashed line represents averaged season length within 8 km of active leks with direct exposure to legal harvest in a given year.

Across the 1995–2018 study period, total area open to hunting declined in most states and provinces (Figs 4 and 5). Hunting season closures were the most direct means to remove harvest pressure and were implemented in greater sage-grouse populations experiencing rapid declines, low population numbers relative to that population’s trend, spatially and numerically small isolated populations and areas on the periphery of larger greater sage-grouse populations, and all Gunnison sage-grouse populations. Over our assessment period, both temporary and long-term hunting closures reduced overall area open to hunting by 30.4% between 1995 and 2018 for greater sage-grouse and 100% for Gunnison sage-grouse. For greater sage-grouse, long-term closures were generally associated with small, isolated populations or areas on the periphery of larger populations (Fig 2). Several individual hunt units representing small portions of larger greater sage-grouse biological populations in Idaho, Montana, Nevada, Oregon, and Utah were closed during the assessment period (Fig 2).

Hunting closures in southwestern Colorado and southeastern Utah preceded the official “threatened” listing decision of Gunnison sage-grouse in 2014 by 14 years. During assessment years, the Gunnison Basin population of Gunnison sage-grouse represented nearly all the species’ exposure to hunting, which ended after the fall hunting season in 1999. This population was exposed to hunting over 9,343 km^2^ between 1995–1999 with a slightly larger area of 9,971 km^2^ in 1998. Note that the 1998 increase in legal area to hunt near the Gunnison sage-grouse range in Colorado was entirely outside of designated Gunnison sage-grouse range. Technically 2.4% of the Piñon Mesa population, representing 0.2% of the total Gunnison sage-grouse distribution, was open to hunting in Utah through the hunting season of 1997. This was an artifact of regulations that defined all of Grand County, Utah open to sage-grouse hunting prior to recognition of Gunnison sage-grouse as a separate species (Fig 2). It is generally believed that very few, if any, hunters were afield in areas with Gunnison sage-grouse in Utah. In addition, Upland Game Reports from Utah between 1995–1997 indicated no take of sage-grouse in southeastern Utah during those years, supporting our contention that Gunnison sage-grouse were unlikely to have been harvested in Utah from 1995–1997.

While permanent closures occurred mainly in small populations and along range periphery for greater sage-grouse (e.g., Canada 1996 and Washington 1988), temporary closures were another common means to reduce hunting pressure. These temporary closures often occurred in response to low populations (i.e., low spring lek counts), wildfire, and West Nile Virus presence. Greater sage-grouse declines at the periphery of the Northern Montana/Canadian and Yellowstone Watershed and Dakotas populations led to temporary closures in North and South Dakota, prior to closures that are not expected to be reopened in the foreseeable future in California (2017); North Dakota (2007); South Dakota (2017); and Jackson, Wyoming (2003; Figs 2 and 3). Additionally, a large portion of the hunted area in Montana (~77% of 1995–2013) was closed to hunting during 2014 and 2015. Disease was a factor in the 2004 hunting closure of the Powder River Basin population in northeastern Wyoming; this population suffered drastic population declines associated with West Nile Virus ([27]; Fig 2), as did parts of southwestern Idaho, though Idaho populations were still open for hunting. Fires in 2012 led to large temporary closures in Nevada and Oregon (Fig 2), while large hunt units in Idaho were closed intermittently due to wildfires between 2008–2016. Research projects examining the effects of harvest contributed to closures in Idaho and a small portion of the Yellowstone Watershed in Montana from 2002–2005 [39, 56] (Fig 2).

While the overall reduction in legal hunting area across the range of greater sage-grouse between 1995 and 2018 was 30.4%, reduction in areas open to harvest within 8 km of an active greater sage-grouse lek was only 16.6% (Fig 4). In other words, direct reduction in potential hunting pressure on greater sage-grouse was much less than total administrative reductions would suggest. Thus, the percent decline of area open to legal greater sage-grouse hunting illustrated that reduction in administrative harvest boundaries did not fully align with a realized reduction in harvest exposure (Fig 4).

### Changes to bag limits, season length, and permit type

**Fig 6:**
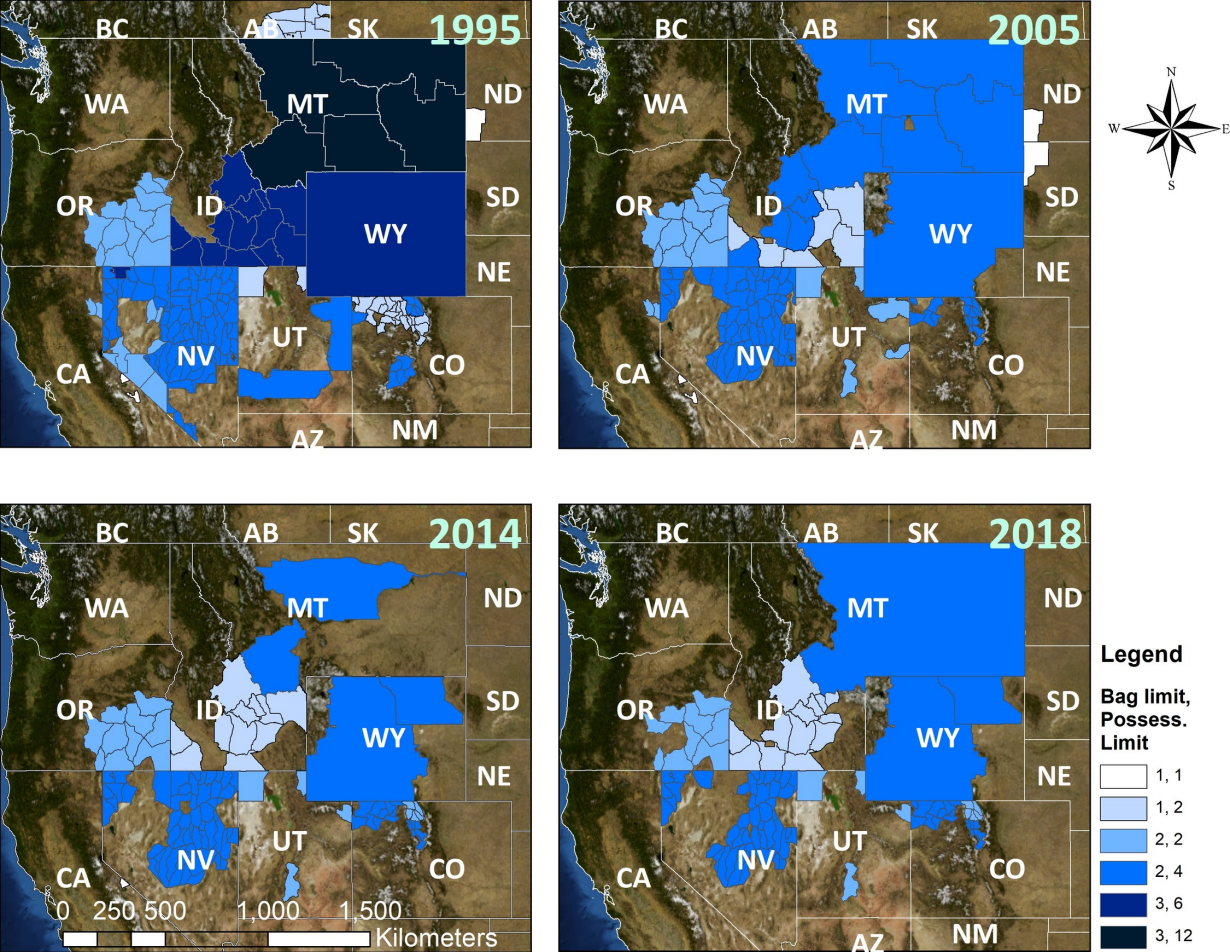
Bag limits, possession limits, and total hunt area for greater sage-grouse in 1995, 2005, 2014, and 2018.

For greater sage-grouse, area-weighted bag limits and possession limits decreased by 39.2% and 52.6%, respectively, across their range between 1995 and 2018 (Figs 5 and 6). The lowest area-weighted possession limits were during 2014 and 2015 when Montana had its most conservative hunting seasons, representing a 45.7% decrease from 1995 to 2015 (Fig 4). Daily bag limits and possession limits ranged from 1–4 and 1–12, respectively, throughout the assessment period. Most hunt areas and years maintained daily bag limits between 1–2 and possession limits between 2–4. Averaged across the range, limits appear to have minimally declined across the study period; however, limits were in fact halved in many areas, from two to one (Table 1). Several hunting units across the greater sage-grouse range had daily bag limits of 1 in numerous years, and North and South Dakota had a possession limit of 1 in years when hunting was open.

This is excluding permit-only hunts, which will be discussed in further detail below. Both bag and possession limits were highest in Montana during our analysis period, where hunters were permitted a possession limit of 12 in 1995 and 6 in several other years. Montana was the only state with a daily bag limit >3, allowing a daily bag of 4 in 2006. Montana, Wyoming, and the hunt associated with Sheldon National Wildlife Refuge and Hunt Unit 33 in Nevada, each had several years with daily bag limits of 3. However, this regulation in Nevada was associated with a permit-only hunt; thus, Nevada limited the overall hunter take by limiting the number of permits. Averaged across the greater sage-grouse range, limits declined across the assessment period. When averaging limits only across areas open to legal harvest (direct exposure to hunting) as opposed to the overall range, these changes appeared smaller due to exclusion of areas without hunting and thereby a 0 daily bag and possession limit (23.3% change in bag limits and 43.1% changes in possession limits from 1995 to 2018; Fig 5).

Changes in daily bag and possession limits for Gunnison sage-grouse were primarily realized in the Gunnison Basin (Colorado) where the daily bag limit was 2 and the possession limit was 4 from 1995–1999. A total of 2.4% of the Piñon Mesa (Utah) population of Gunnison sage-grouse was exposed to 2 and 4, then 1 and 2 daily bag and possession limits during 1995 and 1996–1997, respectively. Utah changed the harvest boundary that overlapped the Gunnison sage-grouse range in southeastern Utah to remove all potential harvest exposure to that group of birds after the 1997 hunting season.

**Fig 7:**
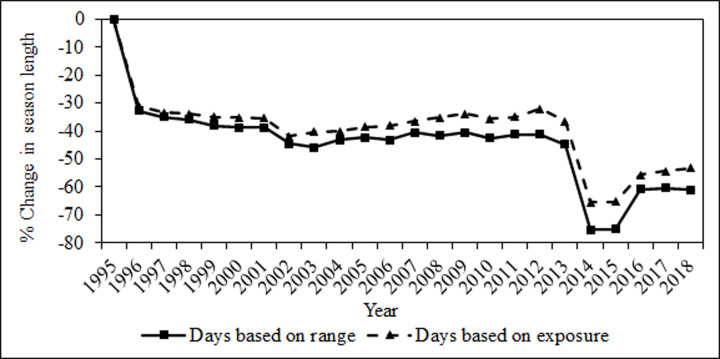
Change over time in season length calculated as total hunt days for greater sage-grouse, 1995–2018. The solid line represents averaged season length within 8 km of all active greater sage-grouse leks within the range of the species, whereas the dashed line represents averaged season length within 8 km of active leks with direct exposure to legal harvest in a given year across the range of greater sage-grouse.

Average season length for greater sage-grouse decreased markedly across the study period: average season length was 32 days in 1995 (range 2–106 days), compared to 12 days in 2018 (range 2–30 days; Fig 7). Montana had the largest influence on the reduction in season length reducing its season from 106 days in 1995 to 62 between 1996–2013 and finally to 30 during 2014–2018. In the Gunnison Basin population of Gunnison sage-grouse, season length was consistently open for 3 weekends, which equated to 17 days in 1995 and 16 days 1996–1999. A small portion of the Piñon Mesa population of Gunnison sage-grouse in Utah was exposed to a 9-day hunting season in Utah from 1995–1997.

Hunt type varied across states, with Colorado, Wyoming, and Montana having solely general season licenses (Box 1). Idaho shifted in 2012 to unlimited sage/sharp-tailed grouse permits to hunt greater sage-grouse and Columbian sharp-tailed grouse, which meant that hunters could pursue both species on the same permit; likely influencing estimated numbers of hunters pursuing either species. California, Oregon, and the hunt associated with Sheldon National Wildlife Refuge and Hunt Unit 33 in Nevada maintained limited permit-only hunting throughout 1995–2018, though California set the permit number to 0 for 2017–2018, effectively ending hunting in the state. Utah shifted from general hunts to permits starting in 2000, with an unlimited number of permits in 2001 and 2002, followed by state-regulated limited permits during 2002–2018. The remainder of states and provinces had some combination of open hunts and permits throughout the assessment period.

### State estimated harvest numbers and hunters

**Fig 8:**
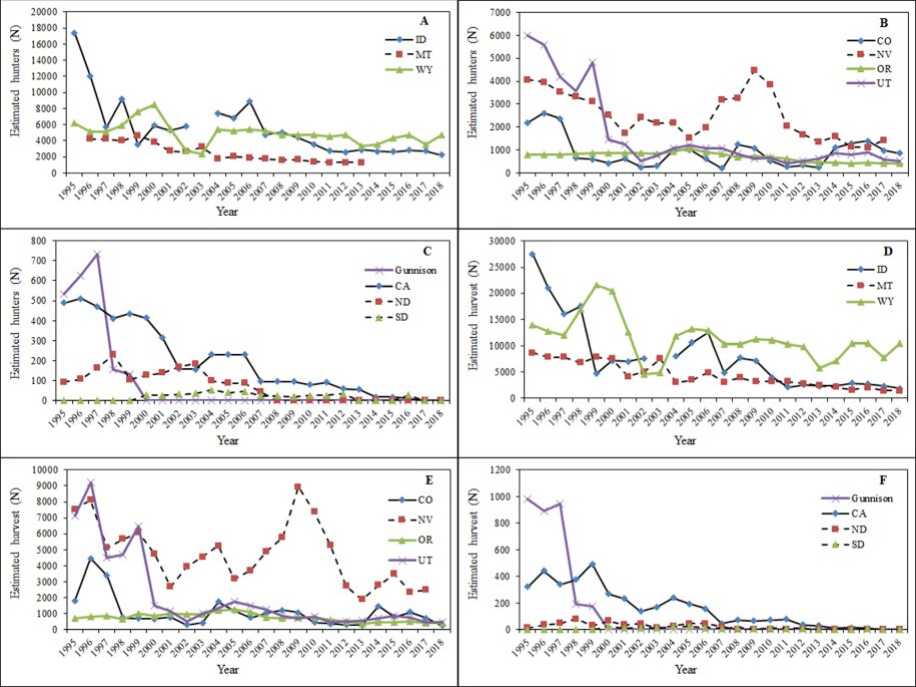
**Estimated number of hunters (A–C) and harvest (D–F) grouped by relative abundance of greater sage-grouse among states from 1995–2018**. Data were obtained from state wildlife management agencies.

Estimated number of harvested greater and Gunnison sage-grouse and hunters afield were available for all years and management agencies with the exception of missing data from Alberta in 1995, Idaho in 2003, and Nevada in 2018 (Fig 8). Total number of hunters declined across all states, and hunter numbers declined by at least 60% in every state except Oregon (declined by 48%) and Wyoming (declined by 24%; Fig 8). Complete cessation of hunting occurred throughout the Gunnison sage-grouse range and in Alberta (1995; final fall of hunting), and several other populations and U.S. states for greater sage-grouse by the end of the assessment period. Previous complete closures for greater sage-grouse by administrative boundary included Saskatchewan (prior to 1950’s and possibly as early as 1916—records prior to 1950 were unavailable) and Washington (1988 onward). The largest total number of hunters was lost from Idaho, where hunter numbers declined by over 15,000 between 1995 and 2018; however, it is difficult to discern what portion of the drop in the number of hunters was from improvements in the hunter survey or real reductions in hunters afield.

Patterns in harvest were generally similar for both species. In 2018, Wyoming had the highest harvest, with over 10,400 birds harvested that year (5-times larger than the next largest state, Idaho; Fig 8D). Annual harvest dipped sharply between 2001 and 2003, and again in 2013 associated with lower population sizes and more conservative season regulations. Another interesting trend was a peak in harvest numbers comparable to pre-1995 numbers in Nevada in 2009 and 2010 and in Oregon throughout much of the mid-2000s (Fig 8E). These increases were related to higher sage-grouse numbers in these years [15, 57]. For Gunnison sage-grouse, the estimated number of hunters and harvested Gunnison sage-grouse from the Gunnison Basin population as verified by upland game reports indicated no harvest in the range of Gunnison sage-grouse within Utah. Estimated number harvested and hunters afield throughout the Gunnison Basin population of Gunnison sage-grouse dramatically decreased from 1997 to 1998, but no apparent hunting regulation changes were linked to this decrease.

## Conclusions and management implications

State and provincial management agencies are tasked with providing hunting opportunities to the public while maintaining the population viability of harvested species. Our summary shows that state and provincial wildlife agencies, in response to scrutiny over declining sage-grouse populations, took extreme efforts to reduce hunting seasons during the 1930s and 1940s and again started a continually more conservative approach to hunting seasons from the mid-1980s, especially 1995, onward. These agencies typically changed harvest regulations, including reducing bag and possession limits, season lengths, and season start date, rather than completely removing hunting exposure to reduce the possibility of additive mortality from hunting on sage-grouse populations while maintaining hunting opportunity for the public. When populations were declining or at risk, state management agencies took steps to eliminate hunting as a threat for those populations, including all Gunnison sage-grouse populations.

Hunting closure, the most dramatic adjustment, was implemented either temporarily or permanently in many harvest management units following declines in greater sage-grouse populations. Studies directly assessing the effectiveness of this technique have been few [39, 56]. Furthermore, disentangling the effects of hunting closure on population growth from expected growth, especially at low population sizes, is challenging [58]. For example, population growth was not detectible after closure of hunting for all Gunnison sage-grouse or greater sage-grouse in Alberta, Canada; Jackson, Wyoming; the bi-state population (California and Nevada); or Washington state populations. However, Connelly et al. [39] found closure of hunting in Idaho was associated with higher spring lek counts for greater sage-grouse, particularly in poorer quality habitat. Compared to closures, reductions in hunting exposure have been a more common method of adjusting harvest numbers for greater sage-grouse. Reductions in exposure have included lowered bag and/or possession limits and shortened or delayed hunting seasons [7]. Such reductions and closures have become increasingly common in the past 25 years, but whether these trends are consistent among management areas or are effective at a larger spatial scale has yet to be documented.

We have presented the most comprehensive summary of these data for multiple reasons. First, varying priorities at the state level and harvest regulations set by state wildlife commissions makes a holistic understanding of sage-grouse hunting exposure range-wide challenging, but we have attempted to provide this view. As a result, we identified some trends that were encouraging and others that might be concerning. First, it appears that overall agencies are doing well with adjusting the timing of hunting seasons, reducing season lengths and maintaining later hunting seasons as supported in the literature [7]. Also, by retaining hunting seasons but lowering bag and possession limits, agencies continue to bring in important funding for conservation from hunting permits while ensuring hunter take is under 5%, as suggested by Reese and Connelly [7], to sustain populations. In addition, constraining hunting seasons rather than closing them is of great importance for state wildlife agencies so they retain the ability to increase hunting opportunity for the public should these populations increase in the future. On the other hand, we identified a trend where apparent reductions in hunt areas are deceptively large; whereas, effective area to harvest sage-grouse (i.e., areas subjected to harvest within 8 km of a lek) declined comparatively less. This may be problematic if one uses total area as an assessment of management effectiveness, giving a false impression of management impacts.

## Supporting information

S1 Table Data for harvest history timeline for sage-grouse from 1870s–2019 stratified by sage-grouse Management Zone and greater and Gunnison sage-grouse populations. All sage-grouse populations that crossed state or provincial boundaries were sub-stratified by those legal boundaries. Green depicts years when a population stratified by state was essentially fully open to hunting, yellow depicts partial closures (>50% open but some closure), orange depicted partial closures (<50% open but some legal hunting), red depicted essentially full hunting closures, and grey with NA indicates years where no data was available.
(XLSX)

S2 Table Data for hunting season regulations for greater sage-grouse. Hunting season regulation and area data for greater sage-grouse across 11 western states and 2 Canadian provinces from 1995–2018. Data used to calculate changes in hunting season regulations for greater sage-grouse relative to the greater sage-grouse range and where sage-grouse were exposed to hunting.
(XLSX)

S3 Table Hunters and harvested sage-grouse data. Estimated number of sage-grouse harvested and number of hunters by year. Data and estimates reported from state and provincial wildlife management agencies.
(XLSX)
